# The 2019 National Nursing Home Social Services Directors Study

**DOI:** 10.1093/geroni/igaa057.2532

**Published:** 2020-12-16

**Authors:** Mercedes Bern-Klug, Robin Bonifas

## Abstract

Psychosocial and emotional needs of nursing home (NH) residents can take the back seat to physical needs in NHs that do not employ staff with the appropriate educational and experience level to anticipate, identify and address these important issues. When present, professional licensed social workers can enhance the NH’s ability to address psychosocial issues. However, federal guidelines do not require licensed social workers in NHs, nor does it collect data that would reveal the extent to which licensed social workers are present. With financial support from the RRF Foundation for Aging the NNHSSD Study was undertaken in 2019 to build understanding of departmental staffing characteristics and involvement of the department in key activities and processes. The 924 respondents from around the country also answered questions about training needs, barriers to addressing resident needs, compensation, and job satisfaction. Findings reveal that about half of the nation’s NH social services directors have earned a bachelors or master’s degree in social work, and about half are licensed (although not all degreed social workers are licensed and not all licensed social workers have earned a social work degree). Half of all social services departments employ only one staff member, one-third have two staff members, and 9% have three staff. About 90% report enjoying their job, with over half reporting they are thriving (not just surviving) in their job. Respondents provided feedback that can be used to strengthen the role of the department and its ability to identity and address resident psychosocial needs.

